# Case of Phthisis, Probably of Contagious Origin

**Published:** 1880-11

**Authors:** 


CASE OF PHTHISIS, PROBABLY OF CONTAGIOUS ORIGIN. Under the care of Dr. MURRELL.)

The chief point of interest in the case was the history, the symptoms having appeared immediately after nursing her husband, who died of phthisis.

Lizzie E., aged twenty-six, was admitted on Sept. 1st, 1879, with phthisis pulmonalis. Her family history was good. Her father, a captain in the navy, was always a very strong man and died of fever abroad. He was not subject to cough, and never, to the best of her knowledge, suffered from any chest complaint. Her mother was living, aged sixty-four years, had been "asthmatical" for the last twenty years. She also had a cough, more or less, all the year round, and was very short of breath if she went out in the fog. The patient had three sisters, all well and healthy. There was no apparent trace of consumption at all in patient's own family.

She was quite well in every way up to the time of her marriage. Her husband, as far as she knew, was a healthy man when she married him, but he came of a consumptive stock. His father died of bronchitis and his mother from phthisis. One of his brothers was, at this time, dying from consumption, and another was far gone with it The patient was married in April, 1878, and in the second week of the following July her husband broke a blood-vessel and lost a great deal of blood. After that he had a cough, grew weaker and weaker, and could hardly do anything.

In August he got too weak to go out, and from that time till the day of his death, in February, 1879, he was assiduously nursed by his wife. He expectorated a great deal, a spitting-cup was of no use to him, and he had to have a hand-basin with water to spit into. Patient hardly ever left him and slept with him. At night he perspired very much, but not enough to wet patient's things, or make her cold. She was in and out of bed pretty well all the night, through the long cold winter of 1878–9, and he refused to have a fire in the room.

After her husband's death the patient was worried and pressed for money, but did not suffer in any way from want of food or comforts. She went to live with her mother and was as well off there as she would have been at home, or perhaps better. She had no cough up to the day of her husband's death; she was then completely knocked up, was seized with cold shivers, which continued for three days. She had a bad cough, and that gradually got worse. Nine months after she had coarse crepitation at both apices, was losing flesh, and sweating profusely at night—was in fact consumptive.

It is certainly a question worth considering whether in cases of advanced consumption with copious expectoration there is not some danger of infection, however slight.

